# Evaluating Mild Cognitive Dysfunction in Patients with Parkinson’s Disease in Clinical Practice in Taiwan

**DOI:** 10.1038/s41598-020-58042-2

**Published:** 2020-01-23

**Authors:** Rwei-Ling Yu, Wei-Ju Lee, Jie-Yuan Li, Yung-Yee Chang, Chin-Chung Chen, Juei-Jueng Lin, Yueh-Feng Sung, Tsu-Kung Lin, Jong-Ling Fuh

**Affiliations:** 1 Institute of Behavioral Medicine, College of Medicine, National Cheng Kung University, Tainan, Taiwan; 2 Institute of Allied Health Sciences, College of Medicine, National Cheng Kung University, Tainan, Taiwan; 3 Neurological Institute, Taichung Veterans General Hospital, Taichung, Taiwan; 4 Faculty of Medicine, National Yang-Ming University Schools of Medicine, Taipei, Taiwan; 5 Institute of Clinical Medicine, National Yang-Ming University Schools of Medicine, Taipei, Taiwan; 6 Department of Neurology, E-Da Hospital, Kaohsiung, Taiwan; 7 School of Medicine, I-Shou University, Kaohsiung, Taiwan; 8 Department of Neurology, Kaohsiung Chang Gung Memorial Hospital, Kaohsiung, Taiwan; 9 Department of Neurology, Shung Ho Hospital, Taipei Medical University, New Taipei City, Taiwan; 10 Department of Neurology, Chushang Show-Chwan Hospital, Nantou, Taiwan; 11 Department of Neurology, Chung-Shan University Hospital, Taichung, Taiwan; 12 Department of Neurology, Tri-Service General Hospital, National Defense Medical Center, Taipei, Taiwan; 13 Department of Neurology, Neurological Institute, Taipei Veterans General Hospital, Taipei, Taiwan

**Keywords:** Parkinson's disease, Human behaviour

## Abstract

Our study aimed to examine the contribution of commonly used tools, including the Mini-Mental State Examination (MMSE) and Montreal Cognitive Assessment (MoCA), and develop a formula for conversion of these tests in the Chinese population. We also create a predictive model for the detection of Chinese patients’ mild cognitive impairment (MCI). We recruited 168 patients with Parkinson’s disease (PD) from 12 medical centres or teaching hospitals in Taiwan, and each participant received a comprehensive neuropsychological assessment. Logistic regression analysis was conducted to find predictors of MCI with the help of a generalized additive model. We found that patients with an MMSE > 25 or a MoCA > 21 were less likely to have MCI. The discrimination powers of the two tests used for detecting MCI were 0.902 and 0.868, respectively, as measured by the area under the receiver operating characteristic curve (ROC). The best predictive model suggested that patients with a higher MMSE score, delayed recall scores of the 12-item Word Recall Test ≥ 5.817, and no test decline in the visuospatial index were less likely to have MCI (ROC = 0.982). Our findings have clinical utility in MCI detection in Chinese PD and need a larger sample to confirm.

## Introduction

Evidence showed that non-motor symptoms are common in patients with movement disorders, Parkinson’s disease (PD)^1^, in which cognitive dysfunction is the most troublesome symptom. The patients’ cognitive profile is heterogeneous, and it is urgently needed to detect the subtle changes of cognitive function before full-brown dementia^2–4^. In the PD population, mild cognitive impairment (MCI) is believed to be a forerunner of dementia, and the prevalence of MCI in PD population is around 26.7% (range: 18.9%–38.2%)^5^. For detecting MCI, diagnostic criteria were suggested by the Movement Disorder Society (MDS), and this criterion has two means to clarify PD patients with MCI. The level I contain the usage of screening tools and the level II involved in the multiple neuropsychological assessments^6^. Clinically, physicians use brief screening tools to measure the patient’s mentality can meet the level I’s requirement. That is, Level I has high clinical utility.

Two most frequently used screening instruments, Mini-Mental State Examination (MMSE)^7^ and the Montreal Cognitive Assessment (MoCA)^8^, applied for screening cognitive dysfunction in patients with Alzheimer’s disease (AD) in clinical practice^9^. However, their clinical utility in the PD population is disputed, especially in the PD population from Eastern countries. MMSE is a universally accepted test and is a handy tool to detect cognitive dysfunction in PD population^10,11^. However, it is prone to ceiling effects and does not provide adequate sensitivity in the detection of executive functions. MoCA is believed to be able to make up for the lack of MMSE and be a more suitable screening instrument for MCI, mild AD, and PD^12^, with acceptable psychometric properties (i.e., good discriminant validity^13^ and reliability^14^). Thus, some researchers suggest that the MoCA, but not the MMSE, is an acceptable tool to measure the MDS Level I criteria^15^. However, the use of this scale is influenced by both various etiologies (AD and PD) and cultures (Western vs. Eastern). Although AD and PD are both neurodegenerative diseases, they have a distinct pathology and cognitive profile. The Taiwanese version of the MoCA has been developed and shows high sensitivity in detecting MCI in patients with AD, although the specificity is not satisfactory^16^. The Taiwanese version of the MMSE has been used widely in the clinical practice of dementia in Taiwan^17^. However, to date, data on the utility of the two tests for patients with MCI in PD (PD-MCI) are not available for Chinese PD populations.

The widespread use of these two tests in multiple clinical centers has called for a standardized method to convert the score of one test to the other forth PD population. One useful conversion formula can integrate data from different centers that use a variety of screening tools. This can enable future research to recruit a more representative sample for early detection and diagnosis of cognitive dysfunction in patients with PD. Converting raw score to Z- or T-score is one way to integrate data from various datasets. Nevertheless, this approach does not consider differences in distribution or variability across populations. Roalf *et al*. (2013) proposed a formula for clinicians to convert the scores of the two screening tests (i.e., MoCA and MMSE) in patients with AD and MCI^18^, but it is not reasonable to apply this formula to the PD population without modification. Recently, investigators proposed a method that was suitable for patients with PD to convert the score of MoCA to MMSE^19^, using the equi-percentile equating method. This approach is performed by converting the raw score to a percentage level and then stratifying, and the same percentage level is considered to be the same category. This method has been revalidated in studies done in the West^20^. This formula provides a conversion table to convert the scores; however, the formula might case the score irregularly distributed. Therefore, it is critical to developing a robust conversion method to detect PD-MCI.

Our main goal was to examine the utility of these two screening tests, MMSE and MoCA, for the currently used PD-MCI criteria in the Chinese PD population. The secondary purpose was to develop a new conversion formula to translate the scores between the two tests, which was specific to the Chinese PD population. The third aim was to create the most efficient regression model to detect Chinese patients with PD-MCI.

## Materials and Methods

### Participants

All the participants (n = 168) were recruited in the Dementia Registry of Parkinson’s Disease (DRPD) study^21^. The patients had been diagnosed as idiopathic PD, following the United Kingdom PD Society Brain Bank clinical diagnostic guideline^22^. Consecutive patients with PD were enrolled through referrals from neurologists of twelve medical centres or teaching hospitals in Taiwan. The inclusion criteria were as follows: idiopathic PD (age at onset > 50 years), no depressive disorder, as indicated by the Geriatric Depressive Scale^23^ score less than 6, and detailed history were collected from caregivers. The following were the criteria for excluding participants: illiteracy, dementia, dementia with Lewy bodies, parkinson-plus syndrome, stroke, have undergone brain surgery, and other systemic or psychiatric diseases (e.g., major depressive disorder) that may affect cognitive function. We also excluded patients who were administered deep brain stimulation operation or medicine (e.g., anticholinergic, cholinesterase inhibitors, or N-Methyl-D-aspartate inhibitors). The demographic and clinical characteristics were collected by interview, and the cognitive function was measured when the patients are in ‘ON’ medication. All participants were informed of the research content and signed the consent form before entering this study, and only the participants who provided their informed consent were allowed to participante in the study, following the 1964 Declaration of Helsinki’s ethical standards. The institutional review boards of each participating hospital (e.g., Shung Ho Hospital, Chushang Show-Chwan Hospital, Chung-Shan University Hospital, Tri-Service General Hospital, Taipei, Taichung, and Kaohsiung Veterans General Hospital, E-Da Hospital, Taipei and Kaohsiung Chang Gung Memorial Hospital, Kaohsiung Medical University Hospital, and Kaohsiung Municipal Ta-Tung Hospital) prove the protocols of the current study. After the data collection procedure, patients were separated into two groups (i.e., cognitively intact patients, CI versus MCI patients) according to the MDS recommendation for diagnosis of PD-MCI^6^. The neurologists judged the patient’s functional dependence and activities of daily life.

### Clinical, neurocognitive, and neuropsychiatric measurement

All patients underwent a neurological assessment to verify the diagnosis of idiopathic PD. The clinical characteristics such as medical history, onset age of PD, and medication data (e,g., levodopa usage) were recorded through a semi-structured interview. In the semi-structured interview, the neurologist asked the patients and their caregivers about their medical history (e.g., diagnosis of any other systemic disease and brain surgery) and clinical characteristics (e.g., onset age, medication, disease severity, and duration). Duration of disease refers to the time since the motor symptoms occurred, as reported by the patient or observed by the caregivers. The Hoehn and Yahr Scale^24^ was applied to measure the disease severity, and the motor disturbances were assessed by neurologists and with the help of part III of the MDS–Unified Parkinson Disease Rating Scale^25^. The patient’s daily levodopa equivalent dosage^26^ was calculated as per the neurologist’s advice.

The general cognitive ability was assessed by the Taiwanese version of MMSE^17^ and the MoCA^16^. Following the MDS recommendation for diagnosis of PD with dementia^10^ or PD-MCI^6^, we evaluated five cognitive domains in detail using the most common clinical tools and criteria. The MDS recorded dysfunction or deficits published criteria or age- and education-matched norm^10,27,28^. Neuropsychiatric features were measured by the Neuropsychiatric Inventory (NPI)^29^, and it contains twelve items. Each symptom’s presence or absence was scored as ‘1’ or ‘0’, respectively. The total NPI score was obtained through the addition of the 12 intensity scores.

### Statistical analysis

The R 3.3.2 software (R Foundation for Statistical Computing, Vienna, Austria) was applied to perform the statistical analysis. An *α* level of 0.05 was set at a statistically significant level. The mean and standard deviation were calculated to describe the distribution of continuous variables, and frequency and percentage were used to depict the categorical variables. The unadjusted effect of the potential prognostic factors or predictors of MCI *versus* cognitively intact (CI) in univariate analysis were tested using Wilcoxon rank-sum and Fisher’s exact test for the data type in patients with PD. The effect size of each test was measured by using Cohen’s *d* (less than 0.49 for small, 0.5 to 0.79 for medium, and large or equal 0.8 for large effect sizes).

The multivariate analysis was performed, and the adjusted effects of prognostic factors or predictors of MCI were identified by a multiple logistic regression model. In our regression analyses, the following model-fitting techniques, including variable selection, the examination of goodness-of-fit (GOF), and diagnosis and correction of regression, were applied. We used the My.stepwise package of R^30^ to performed the stepwise variable selection to acquire the final model. Table 1 showed the univariate relevant covariates (including gender, age, educational years, etc.), and the moderators were selected appropriately. The criteria for entry and stay were set as 0.15, and the final logistic regression model was determined by kick off the covariates whose p-value was larger than 0.05 until the coefficients were different to zero. The independent covariates’ effect in univariate analysis and the mediators’ masking effects in the multivariate analysis might cause the inconsistency between the findings of univariate and multivariate analysis. For example, age and educational level might affect the MMSE total score, and then the latter, in turn, could predict the chance of having MCI. In the current study, the area under the receiver operating characteristic (ROC) curve and the Hosmer-Lemeshow GOF test, as well as the Nagelkerke’s adjusted generalized *R*^2^ were applied to examine the GOF of our model. The value of the ROC larger or equal 0.7 means it has an acceptable distinguishability and the values of Nagelkerke’s adjusted generalized *R*^2^ more significant or equal to 0.30 means an acceptable fitness level for the model. Besides, the Hosmer-Lemeshow GOF test’s *p* values indicate the level of fitness, and the larger, the better.

**Table 1:** Demographic data and clinical characteristics of cognitively intact and mild cognitive impairment PD.

Variable	All PD	PD-CI	PD-MCI	*p*-value
Sample size (*n*)	168	70	98	
Age (year)	69.67 ± 9.48	66.47 ± 9.27	71.96 ± 8.99	<0.001*
Gender, %Male	58.33%	45.92%	54.92%	0.187
Handedness (R/L/B)	163/3/0	69/1/0	94/2/0	0.617
Body Mass Index	23.78 ± 3.24	23.73 ± 3.27	23.81 ± 3.24	0.959
Education, y	9.68 ± 5.35	12.07 ± 4.21	7.91 ± 5.43	< 0.001^*^
Disease duration, y	4.82 ± 4.59	5.38 ± 4.69	4.41 ± 4.50	0.127
levodopa equivalent dose (mg/day)	522.17 ± 418.97	566.32 ± 468.87	490.63 ± 378.71	0.284
H&Y Stages	2.15 ± 0.88	2.14 ± 0.91	2.16 ± 0.87	0.807
**UPDRS**				
part I (mentality)	2.48 ± 1.83	2.55 ± 1.83	2.43 ± 1.85	0.657
part II (daily activities)	9.32 ± 5.89	9.90 ± 5.64	8.90 ± 6.06	0.283
part III (motor)	22.45 ± 12.19	21.44 ± 11.47	23.17 ± 12.69	0.420
part IV	0.99 ± 1.78	1.105 ± 1.94	0.92 ± 1.69	0.997
Mini-Mental State Examination, MMSE	25.54 ± 3.76	28.27 ± 1.54	23.59 ± 3.67	<0.001^*^
MMSE < = 25 (N, %)	71, 42.3%	5, 7.1%	66, 67.3%	—
Montreal Cognitive Assessment, MoCA	21.83 ± 4.99	25.14 ± 3.22	19.46 ± 4.68	<0.001^*^
MoCA< = 21 (N, %)	82, 48.8%	13, 18.6%	69, 70.4%	—
**NPI total score**				
Delusions	0.10 ± 0.43	0.10 ± 0.45	0.10 ± 0.41	0.730
Hallucination	0.11 ± 0.40	0.11 ± 0.46	0.11 ± 0.34	0.528
Agitation/Aggression	0.18 ± 0.56	0.20 ± 0.57	0.17 ± 0.55	0.619
Depression/ Dysphoria	0.51 ± 0.78	0.51 ± 0.73	0.51 ± 0.81	0.688
Anxiety	0.47 ± 0.78	0.57 ± 0.84	0.40 ± 0.74	0.162
Euphoria	0.02 ± 0.20	0.00 ± 0.00	0.05 ± 0.26	0.088
Apathy	0.35 ± 0.73	0.27 ± 0.63	0.41 ± 0.79	0.184
Disinhibition	0.07 ± 0.32	0.07 ± 0.31	0.08 ± 0.34	0.906
Irritability	0.32 ± 0.69	0.32 ± 0.71	0.32 ± 0.68	0.904
Aberrant motor behavior	0.05 ± 0.36	0.08 ± 0.50	0.03 ± 0.22	0.715
Night-time behavior change	0.27 ± 0.67	0.30 ± 0.66	0.25 ± 0.67	0.363
Appetite and eating change	0.11 ± 0.47	0.08 ± 0.40	0.14 ± 0.51	0.243

Data are described as mean ± standard deviation (SD) for continues variables and as number (percentage, %) for categorical variables. Abbreviations: PD, Parkinson’s disease; CI, Cognitively intact; MCI, mild cognitive impairment; BMI, body mass index; UPDRS, Unified Parkinson’s Disease Rating Scale; NPI, Neuropsychiatric Inventory Questionnaire. *p ≤ 0.05 for the statistical testing between PD-CI and PD-MCI groups.

Multiple and straightforward generalized additive models (GAMs) were used to test the non-linear influence of continuous variables and to determine the suitable cut-off point in the stepwise variable selection process. We used vgam function of the VGAM package (Yee, 2017) to fit GAMs for our binary responses in R. The model or data problems were observed by using the detection of influential cases, multicollinearity check and regression diagnostics for residual analysis. The values of variance inflating factor (VIF) mean the happening of the multicollinearity among the covariates in our model. Larger or equal to 10 and 2.5 were applied in continuous and categorical covariates, respectively.

Besides, since the MMSE and the MoCA total scores might not be both available in clinical studies, a new method was developed to make conversions between the total scores of these two crucial screening tools. First, two simple logistic regression models of PD-MCI with the MMSE or MoCA total scores alone were fitted to our data, respectively. Then, the conversion equations were derived based on the proposition that the MMSE or MoCA total scores are considered virtually equivalent if their estimated probabilities of PD-MCI *versus* PD-CI are equal.

## Results

One hundred and sixty-eight patients with PD were recruited in the current study. The number of patients with PD-MCI and PD-CI was 98 and 70, respectively. Table 1 revealed that demographic and clinical data, and the significant differences were found between the two groups for age and educational years. Besides, the cognitive variables and cognitive features were shown in Table 2. For all patients, the most frequent impaired cognitive function was memory; 74.41% of patients have at least one memory test impaired, followed by attention (40.12%) and visuospatial function (38.32%).

**Table 2:** Cognitive variables of cognitively intact and mild cognitive impairment PD.

Variable	All PD	PD-CI	PD-MCI	*p*-value
**Executive Function**				
Category fluency	9.94 ± 2.76	12.53 ± 2.82	10.29 ± 3.25	<0.001
Clock Drawing Test	12.13 ± 4.40	14.81 ± 1.49	10.21 ± 4.78	<0.001
**Executive Index**				
0 test impaired	77.11%	53.13%	46.88%	<0.001
1 test impaired	19.28%	6.25%	93.75%	
2 tests impaired	3.61%	0.00%	100.00%	
**Attention Function**				
Forward score of the Digit Span test	9.41 ± 3.11	10.45 ± 2.67	8.66 ± 3.19	<0.001
Backward score of the Digit Span test	5.78 ± 2.78	6.89 ± 2.06	4.98 ± 2.96	<0.001
Total score of the Digit Span test	15.19 ± 5.21	17.34 ± 3.94	13.65 ± 5.47	<0.001
Sequence subtraction test	3.59 ± 1.65	4.59 ± 0.81	2.89 ± 1.73	<0.001
**Attention Index**				
0 test impaired	59.88%	65.00%	35.00%	<0.001
1 test impaired	34.13%	8.77%	91.23%	
2 tests impaired	5.99%	0.00%	100.00%	
**Memory Function**				
WRT total score	18.79 ± 5.32	21.50 ± 4.05	16.84 ± 5.29	<0.001
WRT delayed recall	5.96 ± 3.01	7.96 ± 1.83	4.53 ± 2.87	<0.001
WRT discrimination index	9.71 ± 2.45	10.78 ± 1.53	8.96 ± 2.70	<0.001
object recall test	1.68 ± 1.20	2.29 ± 0.98	1.25 ± 1.16	<0.001
**Memory Index**				
0 test impaired	25.59%	86.05%	13.95%	<0.001
1 test impaired	49.41%	39.76%	60.24%	
2 tests impaired	25.00%	0.00%	100.00%	
**Visuospatial Function**				
Cube Copying Test	3.05 ± 1.89	4.24 ± 0.89	2.19 ± 1.95	<0.001
Interlocking polygon copy^†^	14.29%	4.17%	95.83%	<0.001
**Visuospatial Index**				
0 test impaired	61.68%	65.05%	34.95%	<0.001
1 test impaired	28.74%	6.25%	93.75%	
2 tests impaired	9.58%	0.00%	100.00%	
**Language Function**				
Naming test	1.99 ± 0.08	2.00 ± 0.00	1.99 ± 0.10	0.398
Repetition test^†^	7.14%	1.43%	11.22%	0.012
**Language Index**				
0 test impaired	92.86%	44.23%	55.77%	0.027
1 test impaired	6.55%	9.09%	90.91%	
2 tests impaired	0.59%	0.00%	100.00%	

Data are shown as mean ± standard deviation (SD) for continues variables and as number (percentage, %) for categorical variables. Abbreviations: PD, Parkinson’s disease; CI, Cognitively intact; MCI, mild cognitive impairment; WRT, 12-item Word Recall Test. ^†^%impaired.

**Figure 1:**
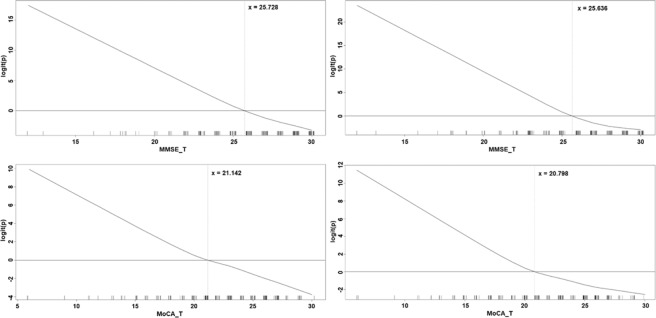
The generalized additive model (GAM) plot for the simple (left) and multiple (right) model of PD-MCI *versus* MMSE (upper) and MoCA (lower). The *X*-axis represents the MMSE-T and the MoCA-T score, and the *Y*-axis is the logit of (probability of PD-MCI). The short vertical lines (i.e., rugs) on the *X*-axis represent the values where patients were located. The range of MMSE and MoCA are 12–30 and 5–30, respectively.

Computing with GAMs by R was conducted for every variable (including age, educational years, and stage, etc.) and resulted in identified PD-MCI. GAMs plots of log (probability of PD-MCI) are shown in Fig. 1. The best prediction combination selected from all of the original and discretized ranges was performed with GLM by R. GAMs plots reveal that the MMSE (or the MoCA) was positively and approximately linearly associated with PD-MCI probability (Fig. 1).

Appropriate cutoff values of the two tests related to the occurrence of PD-MCI were identified by using results from the GAMs. In both GAMs plots for the MMSE, the functional dose of the MMSE intersected the zero PD-MCI probability at a value of 25.728. The sensitivity is 0.659, and the specificity is 0.942, and the Positive Predictive Value (PPV) is 0.935, and the Negative Predictive Value (NPV) is 0.684. In the GAMs plots for MoCA, the intersection point is 21.142. The sensitivity is 0.705, and the specificity is 0.826, and the PPV is 0.838, and the NPV is 0.687.

We used the generalized linear model to predict the best composition from both the original and discretized variables. Table 3

**Table 3:** Multivariate analysis of the predictors for PD-MCI by fitting a multiple logistic regression model with the stepwise variable selection method*.

Covariate**	Estimated Regression Coefficient	Estimated Standard Error	*z* Value	*p* Value	Estimated Odds Ratio	95% Confidence Interval of Odds Ratio	variance inflating factor
Intercept	28.6127	6.2476	4.5798	<0.0001	—	—	—
MMSE^a^ Total Score	−0.9933	0.2219	−4.4772	<0.0001	0.3704	0.2398–0.5721	1.3688
WRT-DR^b^ < 5.817	4.2200	0.9057	4.6597	<0.0001	68.0345	11.5302–401.4398	1.5359
Visuospatial Index: 0 vs. (1 or 2)	−4.5896	1.0837	−4.2350	<0.0001	0.0102	0.0012–0.0850	1.4746

^a^MMSE = Mini-Mental State Examination; ^b^WRT-DR = Delayed Recall Score of the 12-item Word Recall Test. *Goodness-of-fit assessment: *n* = 157, Nagelkerke’s adjusted generalized *R*^2^ = 0.90 > 0.3, the estimated area under the Receiver Operating Characteristic (ROC) curve = 0.982 > 0.7, and the Hosmer and Lemeshow goodness-of-fit *Σ* test *p* = 0.3461 > 0.05 (df = 8), which indicated an excellent fit. **Prediction: To calculate the estimated probability of having PD-MCI (i.e., the *predicted value*, ${\hat{P}}_{i}$) given the observed covariate values of subject *i*, one can use the following formula. According to the above fitted multiple logistic regression model,$$\begin{array}{lll}logit({\hat{P}}_{i}) & = & \log \left(\frac{{\hat{P}}_{i}}{1-{\hat{P}}_{i}}\right)\\  & = & 28.6127+(\,-\,0.9933)\,\times \,{\rm{MMSE}}i\,+\,4.2200\,\times \,{\rm{I}}({\rm{WRT}}-{\rm{DR}}i < 5.817)\\  &  & +\,(\,-\,4.5896)\times \,{\rm{I}}({\rm{Visuospatial}}\,{\rm{Index}}i=0)\end{array}$$

And thus the estimated probability of having PD-MCI for subject *i* is ${\hat{P}}_{i}=\frac{1}{1+\exp [-(28.6127+(-0.9933)\,\times \,{\rm{MMSE}}i+4.2200\,\times \,{\rm{I}}({\rm{WRT}}-{\rm{DR}}i < 5.817)+(-4.5896)\,\times \,{\rm{I}}({\rm{Visuospatial}}\,{\rm{Index}}i=0))]}$

where MMSE = MMSE Total Score, WRT-DR = Delayed Recall Score of the 12-item Word Recall Test, and the indicator function **I**(•) = 1 when the condition • inside the parentheses is true. A conditional effect plot can be drawn accordingly to visualize the estimated probability of having PD-MCI according to the selected covariate values for making predictions in clinical practice.

revealed the results of the multivariate analysis. The variables include the demographics (e.g. age and educational years) and clinical characteristics (e.g., neuropsychological tests). The model demonstrated that the combination of the use of the MMSE, WRT, and the visual-spatial index was the most predictive. The delayed recall score of the 12-item WRT was divided by 5.817 (obtained from the GAMs).

The area under the ROC for the two screening tests and our model was 0.902 (95% confidence interval: 0.856–0.948) for MMSE, 0.868 (95% confidence interval: 0.814–0.922) for MoCA, and 0.982 (95% confidence interval: 0.965–0.999) for our model. The three ROCs displayed no statistically significant difference between the MMSE and MoCA (*p* = 0.197). As expected, our model significantly improved prediction compared to the two screening tests (MMSE, *p* < 0.0001 and MoCA, *p* < 0.0001).

**Figure 2:**
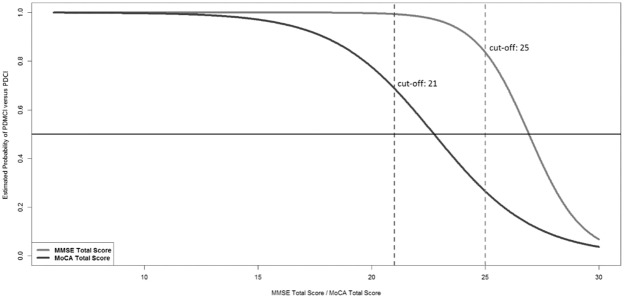
Conditional effect plot of the MMSE (grey) and the MoCA (black) total scores on the estimated probability of PD-MCI. The *X*-axis represents the total scores of tests and the *Y*-axis is the logit of (probability of PD-MCI). The optimal cut-off values of the MMSE and the MoCA were 25 and 21, respectively.

Figure 2 represents the MoCA score with the equivalent MMSE score.

The formula of simple logistic regression models for the MMSE and the MoCA total scores respectively,

$${P}_{1}=\frac{1}{1+{e}^{-({\beta }_{10}+{\beta }_{11}{\rm{MMSE}})}}$$

and

$${P}_{2}=\frac{1}{1+{e}^{-({\beta }_{20}+{\beta }_{21}{\rm{MoCA}})}}$$

where *P* is the probability of PD-MCI versus PD-CI. When setting *P*_1_ = *P*_2_, we obtain

$$\frac{1}{1+{e}^{-({\beta }_{10}+{\beta }_{11}{\rm{MMSE}})}}=\frac{1}{1+{e}^{-({\beta }_{20}+{\beta }_{21}{\rm{MoCA}})}}$$

or

$${e}^{-({\beta }_{10}+{\beta }_{11}{\rm{MMSE}})}={e}^{-({\beta }_{20}+{\beta }_{21}{\rm{MoCA}})}$$

We apply

$${\rm{MMSE}}=\frac{({\beta }_{20}+{\beta }_{21}{\rm{MoCA}})-{\beta }_{10}}{{\beta }_{11}}$$

and

$${\rm{MoCA}}=\frac{({\beta }_{10}+{\beta }_{11}{\rm{MMSE}})-{\beta }_{20}}{{\beta }_{21}}$$

to convert between the total score of MMSE and MoCA, where *β*_10_ = 22.8986, *β*_11_ = −0.8507, *β*_20_ = 10.3083, and *β*_21_ = −0.4531.

## Discussion

Given the importance of effectively detecting cognitive decline in patients with PD, we tested the clinical utility of the MMSE and MoCA screening tools and calculated their optimal cut-off scores for a Chinese PD population. Moreover, a new conversion formula between the two tools in the PD population was developed for clinical use in the current study. Furthermore, we suggest that the combination of the MMSE, the 12-item WRT, and the visuospatial index is the best predictive model for detecting PD-MCI to improve physicians’ diagnostic efficiency.

The cognitive profile of our patients with PD-MCI was compatible with the previous study and showed the language function is relatively preserved^31^. We also found that PD patients’ cognitive function is heterogeneous, and the most vulnerable cognitive function is memory and visuospatial. Our findings were in tune with the evidence that suggests the cognitive impairment is more heterogeneous than those previously appreciated^32^. Moreover, studies suggested that PD patients with posterior cortical dysfunction might have a more rapid disease progression^33^, rather than executive dysfunction^34^. It is worth noting that although there is a group of PD-CI patients who have not reached the diagnostic criteria of PD-MCI, they have several subtle cognitive impairments (i.e., the performance of one test was impaired). This group of patients is worth further research to elucidate whether there is an earlier stage before MCI, for example, pre-MCI stage.

Our main goal was to test the Taiwanese versions of the MoCA and MMSE for the MDS PD-MCI level I criteria. To this end, we recruited patients with PD from twelve medical centres or teaching hospitals and used the MDS Task Force criteria. The diagnostic process of the physician was complicated and required consideration of many factors. For the convenience of clinical use, we identified the optimal cutoff scores from the GAMs plots of the MMSE and MoCA (cut-offs = 25 and 21, respectively). Comparison between level I and II criteria, we used the cut-off score of MMSE and MoCA as the criteria for level I to distinguish PDMCI from PDND. Among the PDMCI patient group classified using level II criteria, 67.3% and 70.4% of them were detected as PDMCI through the level I criteria tool of MMSE and MoCA, respectively. Previous studies provided various cutoff points of MoCA, including 23.5^12^, and 26^13,14^, for PD in different countries. The differing results may be explained by culture, ethnicity, and various PD-MCI diagnostic criteria. Among these reports, one drafted in Singapore^14^ is the most geographically close to Taiwan. Although the two countries are located in the Asia-Pacific region, their culture and language are not similar. China is another country geographically close to Taiwan, and its culture and language are more similar to Taiwan than Singapore. Our results from the Taiwanese versions of the MMSE and MOCA could be used as a reference for the entire Chinese population. Nevertheless, due to the differences in culture and language (e.g. Taiwan used traditional Chinese, and China use Simplified Chinese) between the two countries, it is recommended that one must be cautious with its clinical use and consider the possible effects when interpreting the test result.

In the current study, both MoCA and MMSE have high ROCs (no significant difference was found between their ROCs). Some research reports MMSE as useful in detecting cognitive deterioration in patients with PD^10^, although other studies recommended that MoCA may be the more suitable test^12,13^. Most of these studies had a relatively small sample size and did not apply comprehensive neuropsychological tests, or use various PD-MCI criteria, such as Diagnostic and Statistical Manual of Mental Disorders (5th edition), Petersen’s criteria, or Winblad criteria. Therefore, the application of the above studies may be restricted. Our study suggested that both MMSE and MoCA are suitable for the detection of cognitive dysfunction in Chinese patients with PD. Furthermore, we provided the optimal cut-offs for the detection of PD-MCI in a Chinese population.

A conversion method between various tests will facilitate the comparison and integration of cognitive data from various clinical settings. Van Steenoven and colleagues developed a formula to transform the score of MoCA to an equivalent score of MMSE in patients with PD^19^. Recently, Lawton and colleagues recruited a 10-fold larger cohort of PD patients (*n* = 2,091) to replicate and validate this conversion method. We examined the two published nomograms using our independent PD sample. We found that the Van Steenoven conversion, which has good characteristics (median of 0, small interquartile range, the moderate-to-high relationship between raw and converted MMSE scores and the low proportion of equivalent MMSE scores less than two points different from the raw score), was slightly more precise than the Lawton conversion. Our sample included a more extensive range of MoCA (range: 5–30) performance and education level than previous publications, which enhances the generalizability of this conversion table. Our study confirmed the utility of the conversion table in an Eastern PD population. Furthermore, we developed a new equation for the conversion of the MMSE and MoCA scores, using equiprobability equating and log-linear smoothing. Our straightforward calculation method was developed based on the suggestion that MMSE and MoCA scores can be considered as equivalent if their corresponding probabilities of PD-MCI are equal. We suggest that our equation is a reasonable formula for transforming the score of MoCA to MMSE. Further research is required to validate the formula in an independent PD population.

In addition to the utility of the above two screening tests, we found that the combination of the MMSE, WRT, and the visual-spatial index had the highest predictive value to detect PD-MCI. Neuropsychological deficits are heterogeneous among patients with PD^2^. Our findings highlight the role of visuospatial and memory function in the present study. Past research showed that patients with PD have poor visual-spatial and memory function. Patients with PD scored lower in copying the interlocking pentagons of MMSE than healthy adults, and that visuospatial function is related to the cortical thickness in parietal-temporal regions^35^. However, this result is still controversial^36^. One longitudinal study revealed that the visuospatial ability (i.e., copying intersecting pentagons) has predictive value in the occurrence of dementia in patients with PD^37^. Moreover, free recall memory impairment has been reported to be of the largest magnitude^38^. Whether these deficits are caused by the impairment of encoding or that of retrieval strategy remains controversial. Recently, a study showed that episodic memory function might be related to white matter hyperintense lesions^39^.

The ROC of this model was significantly higher than that of MMSE or MoCA alone. Our findings implied that after taking into account the effects of age, gender, and education, the combination models (usage of the MMSE, WRT, and the visual-spatial index) might be more precise than the might use of MMSE and MoCA to detect patients with PD-MCI. In the current study, we provided a formula for computing predicted values based on the covariates in our fitted multiple logistic regression model. It is noteworthy that the delayed recall score of the WRT was the most potent predictable index. GAMs showed that the WRT score should be divided by 5.817, signifying that patients with PD, with a score of less than 5, have a high probability of PD-MCI. We suggest that this test may be useful when clinicians do not have enough time to implement a comprehensive neuropsychological assessment.

The absence of a matched non-PD control group and follow-up clinical data may be one limitation of this study; nevertheless, the goal of our study was not to explore the cognitive function between participants with and without PD. Evidence suggests that the MoCA and MMSE detect and track cognitive function differently in the PD population. Further work collecting cross-cultural and longitudinal data to validate our findings and to investigate the utility of screening tests is needed. The other limitation is that our sample size was relatively small, and we hope further study can recruit a number of samples to re-validate and confirm the results of this study before generalizing our conclusions.

## Conclusions

We showed different prediction models for PD-MCI: first, using a univariate model with the two screening tests and then by developing a multivariate one. As far as we know, this is the first report to offer the cut-off values of the MMSE and the MoCA for detecting the PD-MCI diagnostic criteria in the Chinese population. In addition, we developed a new conversion formula to transform the score of MoCA to MMSE, in case only one of them is available in clinical practice. We further provided a method to detect PD-MCI through the combined use of the MMSE, WRT, and the visual-spatial index.
